# Pregnancy outcomes among Indian women: increased prevalence of miscarriage and stillbirth during 2015–2021

**DOI:** 10.1186/s12884-023-05470-3

**Published:** 2023-03-08

**Authors:** Periyasamy Kuppusamy, Ranjan K Prusty, Itta K Chaaithanya, Rahul K Gajbhiye, Geetanjali Sachdeva

**Affiliations:** 1 Clinical Research Laboratory, Indian Council of Medical Research - National Institute for Research in Reproductive and Child Health, Mumbai, India; 2 Department of Biostatistics, Indian Council of Medical Research - National Institute for Research in Reproductive and Child Health, Mumbai, India; 3 Department of Molecular Immunology and Microbiology, Indian Council of Medical Research - National Institute for Research in Reproductive and Child Health, Mumbai, India; 4 Cell Physiology and Pathology Laboratory, Indian Council of Medical Research - National Institute for Research in Reproductive and Child Health, Mumbai, India

**Keywords:** Abortion, Miscarriage, Pregnancy loss, Pregnancy outcomes, Stillbirth

## Abstract

**Background:**

Pregnancy outcome is an important health indicator of the quality of maternal health. Adverse pregnancy outcomes is a major public health problem, which can lead to poor maternal and neonatal outcomes. This study investigates the trends in pregnancy outcomes prevalent during 2015–2021 in Indian women.

**Methods:**

The study analysed the data presented in the fourth (2015-16) and fifth (2019-21) rounds of National Family Health Survey (NFHS). The absolute and relative changes in the birth outcomes of last pregnancy during the five years preceding the surveys were estimated using data collected from 195,470 women in NFHS-4 and from 255,549 women in NFHS-5.

**Results:**

Livebirth decreased by 1.3 points (90.2% vs. 88.9%), and nearly half of the Indian states/UTs (n = 17/36) had lower than the national average of livebirth (88.9%) reported during 2019-21. A higher proportion of pregnancy loss was noted, particularly miscarriages increased in both urban (6.4% vs. 8.5%) and rural areas (5.3% vs. 6.9%), and stillbirth increased by 28.6% (0.7% vs. 0.9%). The number of abortions decreased (3.4% vs. 2.9%) among Indian women. Nearly half of the abortions were due to unplanned pregnancies (47.6%) and more than one-fourth (26.9%) of abortions were performed by self. Abortions among adolescent women in Telangana was eleven times higher during 2019-21 as compared to 2015-16 (8.0% vs. 0.7%).

**Conclusion:**

Our study presents evidence of a decrease in the livebirth and an increase in the frequency of miscarriage and stillbirth among Indian women during 2015–2021. This study emphasises that there is a need of regional-specific, comprehensive and quality maternal healthcare programs for improving livebirth among Indian women.

**Supplementary Information:**

The online version contains supplementary material available at 10.1186/s12884-023-05470-3.

## Background

Better maternal health and pregnancy outcomes are significant public health priorities. Adverse pregnancy outcomes such as miscarriage, stillbirth and abortion reflect poor maternal health indicators. Antenatal care (ANC) and institutional delivery are the most important strategies to reduce the higher risk of maternal and fetal complications and deaths. The risk of maternal and neonatal deaths due to complications of pregnancy and childbirth is higher in the low-middle income countries (LMICs) [1]. In India too, approximately 44,000 women die from pregnancy-related complications every year [2]. To improve pregnant women’s health and pregnancy outcomes, the Government of India (GoI) has initiated various programs like *Janani Suraksha Yojana-2005* [3], *Dakshata implementation package-2015* [4], *Pradhan Mantri Surakshit Matritva Abhiyan-2016* [2], *Pradhan Mantri Matru Vandana Yojna-2017* [5], and *LaQshya-2017* [6] to provide a quality of free antenatal check-ups and care during delivery, identify high-risk pregnancies and provide cash incentives.

Miscarriage and stillbirth are the most common natural pregnancy losses, which affects the mother’s physical and psychosocial well-being [7]. Maternal age, abnormal parental genetic makeup, infections, hormonal imbalances, uterine dysfunctions, comorbidities, and lifestyle factors are the attributable risk for higher pregnancy loss, however the cause of miscarriage remains unknown [8]. Patki et al. reported higher prevalence (32%) of spontaneous miscarriages among Indian women in 2016 [9].

India is one of six countries that share half of the global burden of stillbirth [10]. Almost one-third of stillbirth remain unexplained, and two-thirds of cases are reported to be caused by infection in the placenta or umbilical cord, high blood pressure, birth defects, or poor nutrition [8]. To reduce the existing stillbirth rate to 10 per 1000 births by 2030, the Indian New-born Action Plan was implemented in 2014 [11]. There was a substantial reduction in stillbirth rate from 29.6 to 13.9 per 1000 total births during 2000–2019 [10]. The prevalence of stillbirth (4.2 to 14.8) was reported to be widely variable across the Indian states [12]. In recently published study by McClure et al., the major causes of stillbirth were hypertensive diseases (36%), followed by severe anaemia (11%) in Indian and Pakistani population [13]. The authors also reported the maternal and fetal vascular malperfusion in 47% stillbirth as primary placental causes. Intrauterine hypoxia was reported in 72% stillbirth as primary fetal cause of stillbirth [13]. While there has been an improvement in reducing the burden of stillbirth, the pace of this reduction has been slow. This may be partly attributed to the less priority given to stillbirth reduction in national programs. Limited availability of accurate, complete, and actionable information on stillbirth, particularly in high-burden areas, also contributes to slow progress in reducing stillbirth [14, 15].

Unplanned pregnancies are the main reason for seeking abortions [16]. A study estimated that around 15.6 million abortions occurred in India in 2015 [17], and unsafe abortions contribute to 10 to 13% of maternal mortality [18]. Nearly half of the unintended pregnancies ended with abortions and mostly were unsafe [17]. Several factors including socio-cultural barriers contribute to women opting for abortions at outside the healthcare settings. Considering the present needs, *the Medical Termination of Pregnancy (Amendment) Act – 2021* allows universal access to reproductive health services, providing comprehensive abortion care and increasing the upper gestation time limit up to 20 weeks [19]. The study assessed the trends and patterns of pregnancy outcomes across different Indian states and union territories (UTs), considering that several initiatives have been undertaken by the GoI in the last decade to improve maternal health and pregnancy outcomes. It was envisaged that this analysis will directly reflect the impact of various initiatives and also highlight the areas that warrant more efforts towards better maternal healthcare.

## Methods

### Data source and study population

This study was conducted using the nationally representative households survey data of the National Family Health Survey (NFHS) round fourth (2015-16) and fifth (2019-21). Both surveys used probability proportionate sampling, and the methods and data collection tools were published elsewhere [16, 20]. Birth outcomes of last pregnancies among women aged 15–49 years during the five years preceding the survey were considered for analysis. NFHS-4 provides pregnancy outcome data of 1,95,470 women conducted in 29 Indian states and 6 UTs. Recently published NFHS-5 provides pregnancy outcome data of 2,55,549 women conducted in 28 states and 8 UTs. Jammu & Kashmir (J&K) state was divided into two UTs J&K and Ladakh in 2019. The NFHS-4 data for J&K represents both Jammu & Kashmir and Ladakh UTs. Similarly, data of Dadra & Nagar Haveli, and Daman & Diu were reported as one UT during NFHS-5, so we calculated the proportion using the sample size and given proportion of both UTs of pregnancy outcomes in NFHS-4.

### Pregnancy outcome measures

Livebirth is defined as a child born alive. Pregnancy loss refers to pregnancy ending in a non-livebirth due to miscarriage, stillbirth, or abortion. Miscarriage is defined as a pregnancy ended early and involuntarily. Spontaneous abortion or miscarriage refers to fetal death in the womb before 20 weeks of gestation. Stillbirth is defined as birth of a child with no signs of life or fetal demise occurring at the gestation of 28 weeks or later. Abortion is defined as voluntary termination of pregnancy [16].

### Data analysis

Data were extracted from the national and state/UTs- reports of NFHS-4 and NFHS-5. Data of pregnancy outcome measures such as livebirth, pregnancy loss, miscarriage, stillbirth and abortions were reported as proportion (%). The absolute and relative changes were computed. Absolute change refers to the change in the indicator in percentage points i.e. the value of the indicator in NFHS-5 minus that in NFHS-4. Relative change is the absolute change as a percentage of the value of NFHS-4. As per the data available in the report of states and UTs in both surveys, the sub-group analysis was carried out to understand trends in sociodemographic characteristics level. The state-wise map based on the prevalence of the proportion of pregnancy loss was created through ArcGIS 10.1 software packages.

## Results

### Livebirth and pregnancy loss

**Fig. 1:**
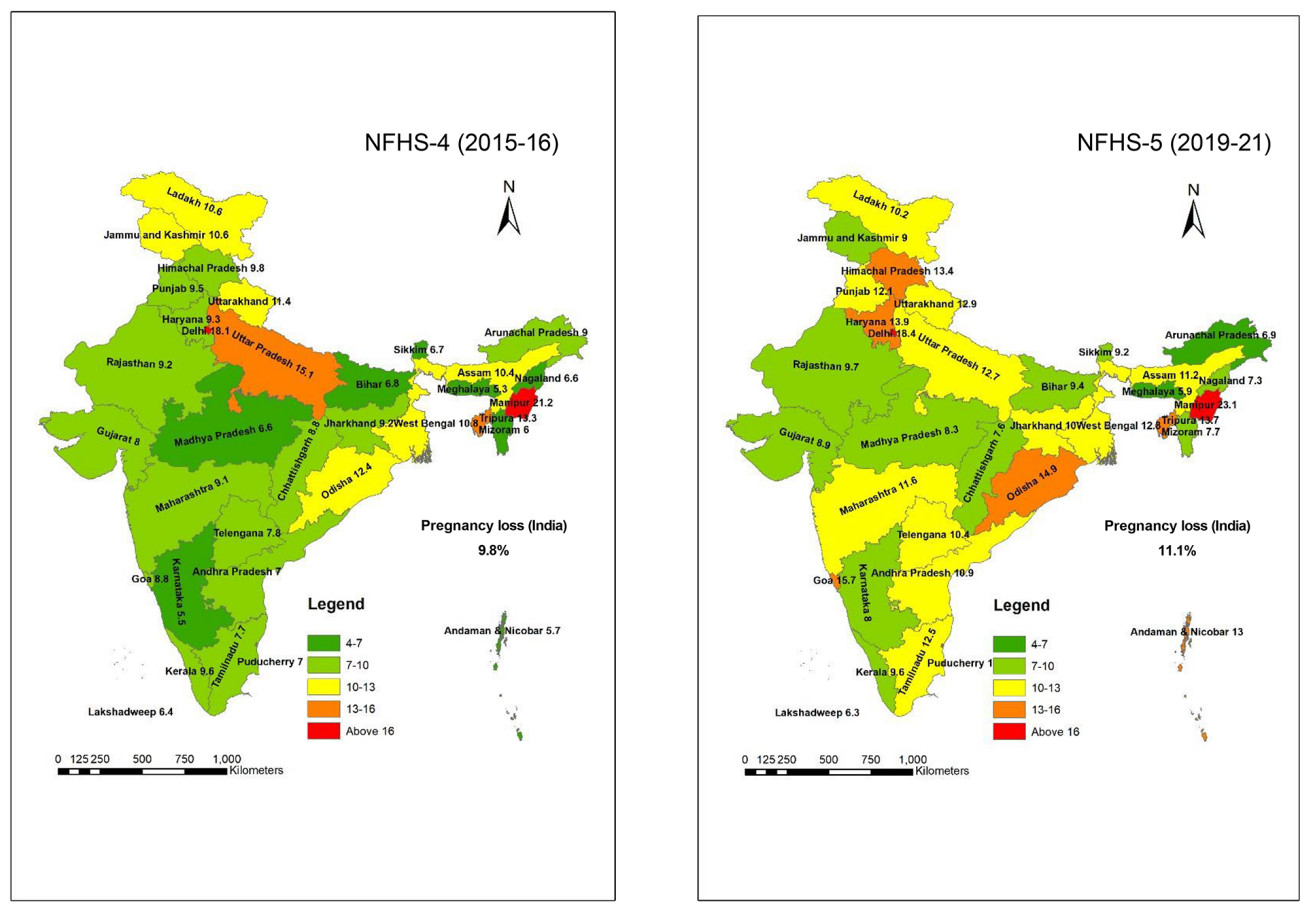
Prevalence of pregnancy loss among Indian women during 2015-21. (Note: Proportion of birth outcomes of the last pregnancy in the five years preceding the survey of women age 15–49; Pregnancy loss includes miscarriage, stillbirth and abortion)

The proportion of livebirth among Indian women was 90.2% (n = 195,470) in 2015-16 and 88.9% (n = 255,549) in 2019-21 (Table 1). Nearly half of the Indian states/UTs (n = 17/36) had lower than the national proportion of livebirth (88.9%) during 2019-21. A trend towards higher pregnancy loss (9.8% ﻿vs. 11.1%) was observed during 2015-21 (Fig. 1). The highest proportion of pregnancy loss (8.5 points) was reported in the UT of Puducherry during 2015-21. Among the Indian states/UTs, the lowest prevalence of livebirth (78.9% vs. 76.8%) and highest pregnancy loss (21.2% ﻿vs. 23.1%) was reported in Manipur. Meghalaya had the lowest proportion of pregnancy loss (5.9%). However, livebirth in Uttar Pradesh increased by 2.4 points (84.9% ﻿vs. 87.3%) and a higher reduction in the pregnancy loss (15.1% ﻿vs. 12.7%) was recorded during 2015-21 (Table 1). About 20.9 points (11.0% ﻿vs. 31.9%) increased proportion of pregnancy loss was found among teenage women in Punjab during 2015-21 (Additional file 1).

**Table 1:** Trends in proportion of livebirth and pregnancy loss across the Indian states/UTs during 2015-21 *

States/UTs	Number of pregnancies		Livebirth				Pregnancy loss				
	NFHS-4	NFHS-5	NFHS-4	NFHS-5	AC	RC	States/UTS	NFHS-4	NFHS-5	AC	﻿RC
Puducherry (PY)	NA	NA	93.0	84.6	-8.4	-9.0	PY	7.0	15.5	8.5	121.4
Andaman & Nicobar Islands (AN)	NA	NA	94.3	87.0	-7.3	-7.7	AN	5.7	13.0	7.3	128.1
Goa (GA)	376	365	91.2	84.3	-6.9	-7.6	GA	8.8	15.7	6.9	78.4
Haryana (HR)	6060	5504	90.8	86.0	-4.8	-5.3	TN	7.7	12.5	4.8	62.3
Tamil Nadu (TN)	6406	5504	92.2	87.5	-4.7	-5.1	HR	9.3	13.9	4.6	49.5
Dadra & Nagar Haveli and Daman & Diu (DD)	NA	NA	92.5	88.3	-4.2	-4.5	DD	7.6	11.7	4.1	53.9
Andhra Pradesh (AP)	2324	2187	93.1	89.0	-4.1	-4.4	AP	7.0	10.9	3.9	55.7
Himachal Pradesh (HP)	2403	2255	90.2	86.6	-3.6	-4.0	HP	9.8	13.4	3.6	36.7
Bihar (BR)	17,499	14,427	93.2	90.6	-2.6	-2.8	BR	6.8	9.4	2.6	38.2
Odisha (OD)	9699	7653	87.7	85.1	-2.6	-3.0	TS	7.8	10.4	2.6	33.3
Punjab (PB)	4449	4865	90.5	87.9	-2.6	-2.9	PB	9.5	12.1	2.6	27.4
Sikkim (SK)	947	551	93.3	90.8	-2.5	-2.7	OD	12.4	14.9	2.5	20.2
Maharashtra (MH)	7379	7879	90.9	88.4	-2.5	-2.8	MH	9.1	11.6	2.5	27.5
Karnataka (KA)	6137	6528	94.5	92.0	-2.5	-2.6	KA	5.5	8.0	2.5	45.5
Telangana (TS)	1882	5768	92.2	89.7	-2.5	-2.7	SK	6.7	9.2	2.5	37.3
Manipur (MN)	4875	2695	78.9	76.8	-2.1	-2.7	WB	10.8	12.8	2.0	18.5
Madhya Pradesh (MP)	18,021	12,028	93.5	91.6	-1.9	-2.0	MN	21.2	23.1	1.9	9.0
West Bengal (WB)	4782	5233	89.2	87.3	-1.9	-2.1	MP	6.6	8.3	1.7	25.8
Uttarakhand (UK)	4617	3385	88.7	87.1	-1.6	-1.8	MZ	6.0	7.7	1.7	28.3
Mizoram (MZ)	3516	1796	93.9	92.3	-1.6	-1.7	UK	11.4	12.9	1.5	13.2
Gujarat (GJ)	6022	7932	92.0	91.1	-0.9	-1.0	GJ	8.0	8.9	0.9	11.3
Assam (AS)	8995	9922	89.6	88.7	-0.9	-1.0	JH	9.2	10.0	0.8	8.7
Jharkhand (JH)	9477	7730	90.7	90.0	-0.7	-0.8	AS	10.4	11.2	0.8	7.7
Nagaland (NL)	3218	2072	93.4	92.7	-0.7	-0.7	NL	6.6	7.3	0.7	10.6
Meghalaya (ML)	3189	4511	94.7	94.1	-0.6	-0.6	ML	5.3	5.9	0.6	11.3
Rajasthan (RJ)	12,590	11,284	90.8	90.2	-0.6	-0.7	RJ	9.2	9.7	0.5	5.4
Tripura (TR)	1263	1939	86.6	86.3	-0.3	-0.3	TR	13.3	13.7	0.4	3.0
Delhi (DL)	NA	2676	81.8	81.6	-0.2	-0.2	DL	18.1	18.4	0.3	1.7
Kerala (KL)	2267	2496	90.4	90.4	0.0	0.0	KL	9.6	9.6	0.0	0.0
Lakshadweep (LD)	NA	NA	93.6	93.6	0.0	0.0	LD	6.4	6.3	-0.1	-1.6
Chandigarh (CH)	NA	NA	84.8	85.1	0.3	0.4	CH	15.1	14.9	-0.2	-1.3
Ladakh (LA)	NA	NA	89.3	89.8	0.5	0.6	LA	10.6	10.2	-0.4	-3.8
Chhattisgarh (CG)	7160	6679	91.1	92.3	1.2	1.3	CG	8.8	7.6	-1.2	-13.6
Jammu & Kashmir (JK)	6313	5041	89.3	91.0	1.7	1.9	JK	10.6	9.0	-1.6	-15.1
Arunachal Pradesh (AR)	4121	4957	91.0	93.2	2.2	2.4	AR	9.0	6.9	-2.1	-23.3
Uttar Pradesh (UP)	31,079	26,947	84.9	87.3	2.4	2.8	UP	15.1	12.7	-2.4	-15.9
**India**	**195,470**	**255,549**	**90.2**	**88.9**	**-1.3**	**-1.4**	**India**	**9.8**	**11.1**	**1.3**	**13.3**

*Proportion of birth outcomes of the last pregnancy in the five years preceding the survey of women age 15–49; Pregnancy loss includes miscarriage, stillbirth and abortion; AC Absolute changes, RC Relative changes; NA Not available

### Miscarriage

The prevalence of miscarriage among Indian women was 7.3% and higher in Manipur (12.3%) in 2019-21. Miscarriage increased in both urban (6.4% ﻿vs. 8.5%) and rural (5.3% ﻿vs. 6.9%) women during 2015-21. An increasing trend in miscarriage was observed in Puducherry (3.4% ﻿vs. 9.9%) (Table 2). Among teenage women, miscarriage was higher in Punjab (23.2%) during 2019-21. Further, miscarriage increased by 4.3 points proportion (2.7% ﻿vs. 7.0%) in the scheduled caste (SC) category in Andhra Pradesh followed by 9.6 points proportion (3.4% ﻿vs. 13.0%) in the scheduled tribes (ST) category in Tamil Nadu and 2.8 points proportion (6.7% ﻿vs. 9.5%) in other backward class (OBC) category in Haryana during 2015-21 (Additional file 2).

**Table 2:** Trends in proportion of pregnancy loss across the Indian States/UTs during 2015-16 and 2019-21 *

Miscarriage					Stillbirth					Abortion				
States/ UTs	NFHS-4	NFHS-5	AC	RC	States/ UTs	NFHS-4	NFHS-5	AC	RC	States/ UTs	NFHS-4	NFHS-5	AC	RC
PY	3.4	9.9	6.5	191.2	AN	0.7	1.8	1.1	157.1	TR	5.1	7.0	1.9	37.3
GA	5.5	10.9	5.4	98.2	LA	0.8	1.5	0.7	87.5	GA	3.3	4.8	1.5	45.5
AN	2.1	6.9	4.8	228.6	TR	0.5	1.1	0.6	120.0	PY	3.6	5.1	1.5	41.7
HR	6.6	10.3	3.7	56.1	HP	0.1	0.6	0.5	500.0	AN	2.9	4.3	1.4	48.3
TN	3.8	7.5	3.7	97.4	OD	0.7	1.2	0.5	71.4	AP	2.9	4.0	1.1	37.9
WB	4.9	8.4	3.5	71.4	ML	0.5	1.0	0.5	100.0	SK	1.1	2.0	0.9	81.8
DD	6.0	9.4	3.4	56.7	PY	0.0	0.5	0.5	NC	HR	1.9	2.7	0.8	42.1
CH	6.4	9.7	3.3	51.6	AS	0.5	0.9	0.4	80.0	TN	3.6	4.4	0.8	22.2
AP	3.4	6.4	3.0	88.2	DL	0.5	0.8	0.3	60.0	TS	3.3	4.1	0.8	24.2
HP	7.2	10.0	2.8	38.9	WB	0.5	0.8	0.3	60.0	KA	1.8	2.4	0.6	33.3
PB	6.1	8.4	2.3	37.7	DD	0.0	0.3	0.3	NC	DD	1.5	2.0	0.5	33.3
MN	10.0	12.3	2.3	23.0	TN	0.3	0.6	0.3	100.0	PB	2.7	3.1	0.4	14.8
MH	4.9	7.1	2.2	44.9	UK	0.9	1.1	0.2	22.2	BR	1.3	1.7	0.4	30.8
MZ	5.3	7.4	2.1	39.6	MP	0.6	0.8	0.2	33.3	HP	2.5	2.8	0.3	12.0
BR	4.6	6.6	2.0	43.5	BR	0.9	1.1	0.2	22.2	MH	3.8	4.0	0.2	5.3
OD	7.0	9.0	2.0	28.6	JH	1.0	1.2	0.2	20.0	LD	1.7	1.9	0.2	11.8
KA	3.2	5.1	1.9	59.4	TS	0.4	0.6	0.2	50.0	UK	3.3	3.4	0.1	3.0
MP	4.4	6.2	1.8	40.9	HR	0.8	0.9	0.1	12.5	OD	4.7	4.7	0.0	0.0
TS	4.1	5.7	1.6	39.0	RJ	0.6	0.7	0.1	16.7	MZ	0.2	0.2	0.0	0.0
SK	4.8	6.3	1.5	31.3	SK	0.8	0.9	0.1	12.5	JH	2.6	2.4	-0.2	-7.7
KL	4.7	6.2	1.5	31.9	GJ	0.5	0.6	0.1	20.0	GJ	2.2	2.0	-0.2	-9.1
DL	10.5	11.9	1.4	13.3	MH	0.4	0.5	0.1	25.0	LA	3.5	3.2	-0.3	-8.6
UK	7.2	8.4	1.2	16.7	JK	0.8	0.8	0.0	0.0	MP	1.6	1.3	-0.3	-18.8
NL	4.1	5.2	1.1	26.8	CG	1.0	1.0	0.0	0.0	NL	2.0	1.7	-0.3	-15.0
ML	3.6	4.7	1.1	30.6	MN	0.4	0.4	0.0	0.0	MN	10.8	10.4	-0.4	-3.7
AS	4.4	5.5	1.1	25.0	GA	0.0	0.0	0.0	NC	RJ	2.0	1.5	-0.5	-25.0
GJ	5.3	6.3	1.0	18.9	KA	0.5	0.5	0.0	0.0	CG	2.4	1.7	-0.7	-29.2
RJ	6.6	7.5	0.9	13.6	PB	0.7	0.6	-0.1	-14.3	AS	5.5	4.8	-0.7	-12.7
JH	5.6	6.4	0.8	14.3	NL	0.5	0.4	-0.1	-20.0	ML	1.2	0.2	-1.0	-83.3
LD	3.3	4.1	0.8	24.2	KL	0.3	0.2	-0.1	-33.3	JK	3.5	2.3	-1.2	-34.3
UP	8.6	8.5	-0.1	-1.2	AR	0.6	0.4	-0.2	-33.3	AR	4.0	2.7	-1.3	-32.5
JK	6.3	5.9	-0.4	-6.3	AP	0.7	0.5	-0.2	-28.6	DL	7.1	5.7	-1.4	-19.7
CG	5.4	4.9	-0.5	-9.3	UP	1.4	1.1	-0.3	-21.4	KL	4.6	3.2	-1.4	-30.4
AR	4.4	3.8	-0.6	-13.6	CH	1.4	1.0	-0.4	-28.6	WB	5.4	3.6	-1.8	-33.3
LA	6.3	5.5	-0.8	-12.7	MZ	0.5	0.1	-0.4	-80.0	UP	5.1	3.1	-2.0	-39.2
TR	7.7	5.6	-2.1	-27.3	LD	1.4	0.3	-1.1	-78.6	CH	7.3	4.2	-3.1	-42.5
**India**	**5.7**	**7.3**	**1.6**	**28.1**	**India**	**0.7**	**0.9**	**0.2**	**28.6**	**India**	**3.4**	**2.9**	**-0.5**	**-14.7**

*Proportion of birth outcomes of the last pregnancy in the five years preceding the survey of women age 15–49;* AC-Absolute changes, RC-Relative changes; NC-Not calculated*

### Stillbirth

The prevalence of stillbirth in India was 0.9% during 2019-21 and it relatively increased by 28.6%. About 1.1 points proportion (0.7% ﻿vs. 1.8%) of stillbirth increased in Andaman & Nicobar Islands during 2015-21 (Table 2). Among different age groups, there was a higher prevalence of stillbirth noted among 15–19 years in Madhya Pradesh (2.0%). Stillbirth prevalence increased by 2.1% in the age group of women in 20–39 years in Sikkim and 5.2% in 40–49 years-old women in West Bengal, as compared with other Indian states/UTs during 2019-21. The prevalence of stillbirth was higher among women in rural than urban (0.9% vs. 0.7%), women with no education than highly educated (1.1% vs. 0.6%) and in women belonging to SC than ST and OBC (1.0% vs. 0.8%) categories observed during 2019-21 (Additional file 3).

### Abortion

Overall, the frequency of abortions declined up to 15% (relative changes) among Indian women during 2015-21. The prevalence was higher than the national average (2.9%) in Manipur (10.4%) during 2019-21. On the other hand, Meghalaya and Mizoram (0.2% each) had the lowest proportion of abortions in 2019-21. Abortions increased by 1.9 points proportion (5.1% ﻿vs. 7.0%) in Tripura and the highest decline was observed in Chandigarh (7.3% ﻿vs. 4.2%) during 2015-21 (Table 2). However, an eleven-fold increase in abortion was noted among teenage pregnancies in Telangana (0.7% ﻿vs. 8.0%) during 2015-21. More abortions were reported in urban women than in rural (4.0% vs. 2.5%) during 2019-21. (Additional file 4).

Women undergoing abortion at public health hospitals in Kerala (20.9% ﻿vs. 48.5%) and private health facilities in Himachal Pradesh sharply increased during 2015-21. In India, more than half of the abortions were performed at private health sector (52.4% ﻿vs. 52.9%) than in public health sector (20.2% ﻿vs. 20.3%) during 2015-21. Women performing abortions at home increased by 21 points proportion (13% ﻿vs. 34%) in Punjab and 19.9 points proportion (18.7% ﻿vs. 38.6%) in Rajasthan during 2015-21. About more than half (55.7%) of women in Odisha and one-quarter (26.2%) of women in India aborted their foetuses at home during 2019-21 (Additional file 5). Half of the abortions (54.8%) were performed by doctors followed by 13.5% by nurses or auxiliary nurse midwives or lady health visitors and 26.9% by self in India during 2019-21. However, a higher proportion of self-abortions was noted in Odisha (54.1%) and lower proportion in Telangana (4.8%) during 2019-21 (Additional file 6). Among various reasons for seeking an abortion, the most commonly reported were unplanned pregnancies (47.6%), health did not permit (11.3%), the last child being too young (9.7%), and pregnancy complications (9.1%) during 2019-21. Abortions due to unplanned pregnancy (73.5% in Delhi), last child being too young (24.9% in Chhattisgarh), complications in pregnancy (26.2% in Punjab), and congenital abnormalities (16% in Kerala) were higher (Additional file 7).

## Discussion

The present study highlights a trend towards decrease in the proportion of livebirth during 2015–2021. The proportion of livebirth was 88.9% during 2019-21 among Indian women, which is comparatively lower than reported in other low-middle income countries like Ghana (95.1%), Democratic Republic of the Congo (97.1%), Zambia (99.2%), and Kenya (99.9%) [1] and higher than the Ethiopian population (84.1%) [21]. The data from the last two rounds of NFHS showed that livebirth declined by 1.3 point percent from 2015-16 to 2019-21. Livebirth proportion was lower in seventeen Indian states/UTs as compared to the national level in 2019-21. Despite launch of various programs and schemes by the GoI for improving maternal health and outcomes, a trend towards the reduction in livebirth proportion was observed in many states [2–6]. Age at conception, mode of conception and psychological well-being during pregnancy are the major determinants of a livebirth [22]. In addition, other factors such as anemia, infection, hypertension, hyperglycemia, spousal violence, and environmental pollution also contribute to high pregnancy losses [23, 24]. Further inequality of socioeconomic status in urban and rural areas among the Indian states/UTs might be one of the factors for the reduction of livebirth rates during 2019–21 [25].

The highest reduction of livebirth among teenage women was observed in Punjab. A report shows 2.6% of teenage girls in Punjab became pregnant [20] and also a higher rate of pregnancy loss was reported in vulnerable populations [26]. Child marriage could result in teenage pregnancy due to social pressure, low education, and lack of knowledge about sexual and reproductive health. Teenage pregnancies pose a serious health risk to both mother and fetus and were also higher risks for miscarriage, preterm birth, low birth weight, and intrauterine growth retardation [27]. The *Prohibition of Child Marriage (Amendment) Act, 2021* enables raising women’s marriage age from 18 to 21 years, which could bring down the incidence of teenage pregnancies [28]. Reduction of teenage pregnancy through community awareness not only improves women’s reproductive health but also lowers the incidence of miscarriage and stillbirth.

The proportion of pregnancy loss increased by 1.3 points proportion among Indian women. The pregnancy loss reported in our study (11.1%) during 2019-21, was higher than Malawi (0.6%), South Africa (2.5%), Uganda (1.4%) and Zimbabwe (1%) [29]. Higher proportion of pregnancy loss was noted in some of the states like Manipur, Odisha, Haryana, Himachal Pradesh, and Tripura during 2019-21. Further, there are states/UTs like Puducherry, Andaman and Nicobar Islands, Goa, and larger states like Tamil Nadu and Haryana where pregnancy loss increased during 2019-21 as compared to 2015-16. Pregnancy loss can be prevented by increasing access to high-quality healthcare services in the public health sector. Although, the WHO recommends at least eight ANC visits during pregnancy, only 58% of Indian mothers received 4 ANC visits during their last childbirth [16]. Higher frequency of ANC visits is associated with lower chance of pregnancy loss [12]. Indian women who had maternal hypertension, antepartum haemorrhage, short gestation age, and asphyxia during labor are reported to experience pregnancy loss [30]. The other risk factors for pregnancy loss were poor nutrition [31] and spousal violence, which may cause anxiety and depression [16]. The prevalence of miscarriage was 73 per 1000 pregnancies and relatively increased by 28.1% among Indian women. This prevalence was higher than that reported in neighbouring countries like Pakistan (3.6%) and Bangladesh (5.8%) [1]. It was higher particularly in teenage, older, highly educated, and urban women.

Miscarriage leads to a physical risk of bleeding or infections and a psychological risk of anxiety, depression, and post-traumatic stress [7]. The known predisposing risk factors were low body mass index, anemia, overweight and obesity, hypertension, and diabetes [32] and it was higher among advanced maternal-aged women [33] and also in educated urban women [34]. The prevalence of stillbirth increased from 0.7 to 0.9% among Indian women, however it was lower than reported in Malawi (2.3%) and Uganda (3.0%) [29]. Apart from medical factors, stillbirth is also associated with various social factors such as vulnerability based on place of residence  [35], and low socioeconomic status [12].

Overall, the reported incidence of abortion decreased by 15%. It could be the impact of the COVID-19 pandemic, which resulted in the disruption of abortion care services [36]. However, eleven fold higher proportion of abortion was noted among adolescent women in Telangana. Several abortions are driven by socioeconomic vulnerability and demographic determinants including wealth quintiles, maternal age, education, and lack of awareness on the use of contraceptive methods [18, 37]. A higher prevalence of self-abortion was recorded in Odisha, Tripura, Arunachal Pradesh, Chhattisgarh and Bihar, where women with socioeconomic vulnerability, hard-to-reach healthcare settings and social stigma together pose women at higher risk for unsafe abortion [18]. In India, two-thirds of the population lives in rural settings, due to inadequate health care and transport facilities, more abortion-related deaths are reported rural areas of India [38]. A recent study shows that around 10% of maternal deaths in India were due to abortions [18].

Our study has few limitations. This study is the compilation of the reports of national and states/UTs, the unit-level data is not utilized for this study. Some data for UTs were not available in the report and the cross-sectional study doesn’t give any causal relationship. Further, there were some UTs boundary change during the study period, we have used aggregate proportions for comparison.

### Policy recommendations

 Increase prevalence of miscarriage in both urban and rural areas is a matter of concern. The government may focus in improving the health infrastructure in rural and underprivileged areas.

The stillbirth rate is an indicator of the quality and equity of health care. Increased stillbirth leads to the heavy burden of psychosocial and economic costs to the family as well as to the country [39]. The government should ensure respectful maternity services including bereavement care as majority of the women experience various psychological symptoms after the death of their baby. The health system improvement may reduce the incidence of preventable stillbirth, therefore high quality antenatal and intrapartum care should be provided through public health care system in India.

Our study reported nearly half of abortions were due to unplanned pregnancies. These findings strongly suggest the need for more research on interventions that improve the knowledge and practice of providing medical abortion in India. A priority should be given for improving policies and practices at national and state level to increase access to comprehensive abortion care and quality contraceptive services for preventing unplanned pregnancies.

## Conclusion

This study found that the prevalence of miscarriage and stillbirth increased in many Indian states/UTs during 2015-21. Antenatal health check-ups have gone up but still, there is evidence of low livebirth in some states/UTs. Hence, the quality of ANC check-ups needs greater attention in the health mission programs. Miscarriage contributes a major share of pregnancy loss. Controllable factors such as maternal weight, hypertension, anemia and blood sugar should be paid a greater attention in prevent miscarriage and stillbirth in India. Although, abortion rate is low, the major concern is around half of the abortions are not done by qualified medical professionals. Prevention of unsafe abortion practices in some of states requires the highest priority.

## Electronic supplementary material

Below is the link to the electronic supplementary material.

**Supplementary Material 1**

## Data Availability

The data used for the study is publicly available from the Demographic and Health Surveys Program (https://dhsprogram.com/Countries/Country-Main.cfm?ctry_id=57&c=India). However, the dataset(s) supporting the conclusions of this study is included within the article and its additional files.
