# Supplementary material for: Pregnancy outcomes among Indian women: increased prevalence of miscarriage and stillbirth during 2015–2021
Source: BMC Pregnancy Childbirth. 2023 Mar 8;23:150. doi: 10.1186/s12884-023-05470-3 (PMC9992916; doi:10.1186/s12884-023-05470-3)
Supplement: Supplementary file 1 — Supplementary Material 1 [file 12884_2023_5470_MOESM1_ESM.docx]

**Supplementary File (Appendix)**

| **Additional file 1: Changes in proportion of live birth among different age groups across the States/UTs during 2015-16 and 2019-21 *** | | | | | | | | | | | | | | | | | | |  |
| --- | --- | --- | --- | --- | --- | --- | --- | --- | --- | --- | --- | --- | --- | --- | --- | --- | --- | --- | --- |
| **Age of 15-19** | | | | | **Age of 20-29** | | | | | **Age of 30-39** | | | | | **Age of 40-49** | | | | |
| States /UTs | NFHS-4 | NFHS-5 | AC | RC | States /UTs | NFHS-4 | NFHS-5 | AC | RC | States /UTs | NFHS-4 | NFHS-5 | AC | RC | States /UTs | NFHS-4 | NFHS-5 | AC | RC |
| PB | 89.0 | 68.1 | -20.9 | -23.5 | GA | 92.9 | 80.3 | -12.6 | -13.6 | BR | 92.9 | 88.6 | -4.3 | -4.6 | WB | 60.0 | 80.0 | 20.0 | 33.3 |
| TS | 94.6 | 75.8 | -18.8 | -19.9 | MN | 83.6 | 80.2 | -3.4 | -4.1 | AP | 92.7 | 89.0 | -3.7 | -4.0 | TR | 69.5 | 78.1 | 8.6 | 12.4 |
| HR | 84.3 | 74.7 | -9.6 | -11.4 | HR | 91.4 | 88.8 | -2.6 | -2.8 | HR | 89.4 | 86.3 | -3.1 | -3.5 | JM | 81.0 | 89.4 | 8.4 | 10.4 |
| TN | 91.5 | 84.8 | -6.7 | -7.3 | TS | 93.9 | 91.7 | -2.2 | -2.3 | MH | 90.6 | 87.9 | -2.7 | -3.0 | KA | 81.7 | 86.5 | 4.8 | 5.9 |
| KA | 90.7 | 84.3 | -6.4 | -7.1 | PB | 91.1 | 89.0 | -2.1 | -2.3 | MP | 93.3 | 91.0 | -2.3 | -2.5 | AR | 91.1 | 94.7 | 3.6 | 4.0 |
| UK | 79.2 | 73.2 | -6.0 | -7.6 | BR | 93.9 | 91.8 | -2.1 | -2.2 | MZ | 94.9 | 92.6 | -2.3 | -2.4 | UP | 78.4 | 80.5 | 2.1 | 2.7 |
| HP | 89.6 | 84.7 | -4.9 | -5.5 | TN | 93.3 | 91.4 | -1.9 | -2.0 | SK | 92.5 | 90.5 | -2.0 | -2.2 | MZ | 83.1 | 84.9 | 1.8 | 2.2 |
| MP | 87.8 | 83.6 | -4.2 | -4.8 | NL | 95.7 | 93.8 | -1.9 | -2.0 | TN | 89.6 | 87.9 | -1.7 | -1.9 | JH | 86.0 | 87.4 | 1.4 | 1.6 |
| NL | 92.4 | 88.5 | -3.9 | -4.2 | TR | 90.1 | 88.6 | -1.5 | -1.7 | KA | 93.2 | 91.7 | -1.5 | -1.6 | KL | 80.4 | 81.3 | 0.9 | 1.1 |
| MH | 86.1 | 82.7 | -3.4 | -3.9 | AP | 93.8 | 92.3 | -1.5 | -1.6 | WB | 84.5 | 83.4 | -1.1 | -1.3 | OD | 79.9 | 78.3 | -1.6 | -2.0 |
| ML | 95.6 | 92.4 | -3.2 | -3.3 | KA | 95.2 | 93.8 | -1.4 | -1.5 | OD | 85.5 | 84.5 | -1.0 | -1.2 | CG | 89.0 | 87.3 | -1.7 | -1.9 |
| AR | 90.2 | 88.9 | -1.3 | -1.4 | OD | 89.2 | 88.2 | -1.0 | -1.1 | UK | 88.2 | 87.4 | -0.8 | -0.9 | PB | 85.9 | 84.1 | -1.8 | -2.1 |
| AS | 90.0 | 89.4 | -0.6 | -0.7 | MP | 93.9 | 93.3 | -0.6 | -0.6 | MN | 76.6 | 75.9 | -0.7 | -0.9 | ML | 92.8 | 90.8 | -2.0 | -2.2 |
| AP | 85.0 | 84.6 | -0.4 | -0.5 | WB | 90.9 | 90.8 | -0.1 | -0.1 | GJ | 90.9 | 90.5 | -0.4 | -0.4 | GJ | 86.3 | 84.1 | -2.2 | -2.5 |
| GJ | 86.3 | 86.0 | -0.3 | -0.3 | MH | 91.3 | 91.3 | 0 | 0 | JH | 89.9 | 89.6 | -0.3 | -0.3 | NL | 90.5 | 86.2 | -4.3 | -4.8 |
| BR | 86.1 | 86.1 | 0 | 0 | AS | 90.9 | 90.9 | 0 | 0 | NL | 91.6 | 91.5 | -0.1 | -0.1 | HR | 87.8 | 83.2 | -4.6 | -5.2 |
| WB | 90.6 | 91.3 | 0.7 | 0.8 | JH | 91.7 | 91.8 | 0.1 | 0.1 | AS | 87.5 | 87.8 | 0.3 | 0.3 | RJ | 90.5 | 85.7 | -4.8 | -5.3 |
| MZ | 92.5 | 93.9 | 1.4 | 1.5 | UK | 89.1 | 89.2 | 0.1 | 0.1 | HP | 89.1 | 89.7 | 0.6 | 0.7 | TN | 82.9 | 77.0 | -5.9 | -7.1 |
| CG | 80.7 | 82.7 | 2.0 | 2.5 | MZ | 94.9 | 95.1 | 0.2 | 0.2 | ML | 94.0 | 95.0 | 1.0 | 1.1 | MP | 90.1 | 84.2 | -5.9 | -6.5 |
| OD | 84.4 | 86.8 | 2.4 | 2.8 | HP | 90.8 | 91.1 | 0.3 | 0.3 | PB | 89.6 | 90.7 | 1.1 | 1.2 | AS | 82.6 | 76.3 | -6.3 | -7.6 |
| RJ | 80.6 | 83.7 | 3.1 | 3.8 | RJ | 91.5 | 91.9 | 0.4 | 0.4 | RJ | 90.0 | 91.4 | 1.4 | 1.6 | HP | 86.1 | 77.4 | -8.7 | -10.1 |
| TR | 87.6 | 91.4 | 3.8 | 4.3 | ML | 95.5 | 96.0 | 0.5 | 0.5 | GA | 89.8 | 91.4 | 1.6 | 1.8 | BR | 92.5 | 83.2 | -9.3 | -10.1 |
| JH | 85.5 | 90.4 | 4.9 | 5.7 | GJ | 92.9 | 93.5 | 0.6 | 0.6 | CG | 90.4 | 92.1 | 1.7 | 1.9 | MN | 66.6 | 54.2 | -12.4 | -18.6 |
| UP | 71.8 | 77.9 | 6.1 | 8.5 | SK | 93.6 | 94.9 | 1.3 | 1.4 | JK | 88.8 | 90.5 | 1.7 | 1.9 | MH | 87.4 | 73.1 | -14.3 | -16.4 |
| JK | 91.0 | 100.0 | 9.0 | 9.9 | AR | 92.1 | 93.9 | 1.8 | 2.0 | KL | 90.5 | 93.2 | 2.7 | 3.0 | GA | 87.9 | 69.3 | -18.6 | -21.2 |
| MN | 81.4 | 92.0 | 10.6 | 13.0 | KL | 90.8 | 92.7 | 1.9 | 2.1 | UP | 82.6 | 85.7 | 3.1 | 3.8 | UK | 86.7 | 65.3 | -21.4 | -24.7 |
|  |  |  |  |  | UP | 87.0 | 88.9 | 1.9 | 2.2 | TS | 86.5 | 90.2 | 3.7 | 4.3 |  |  |  |  |  |
|  |  |  |  |  | CG | 91.7 | 93.9 | 2.2 | 2.4 | AR | 89.4 | 93.3 | 3.9 | 4.4 |  |  |  |  |  |
|  |  |  |  |  | JK | 90.5 | 93.5 | 3.0 | 3.3 | TR | 78.6 | 83.8 | 5.2 | 6.6 |  |  |  |  |  |
| ** Proportion of birth outcomes of the last pregnancy in the five years preceding the survey of women age 15-49; AC-absolute changes, RC-Relative changes* | | | | | | | | | | | | | | | | | | |  |

| **Additional file 2: Changes in proportion of miscarriage among different community levels across the States/UTs during 2015-16 and 2019-21 *** | | | | | | | | | | | | | | |
| --- | --- | --- | --- | --- | --- | --- | --- | --- | --- | --- | --- | --- | --- | --- |
| **SC** | | | | | **ST** | | | | | **OBC** | | | | |
| States /UTs | NFHS-4 | NFHS-5 | AC | RC | States /UTs | NFHS-4 | NFHS-5 | AC | RC | States /UTs | NFHS-4 | NFHS-5 | AC | RC |
| AP | 2.7 | 7.0 | 4.3 | 159.3 | TN | 3.4 | 13.0 | 9.6 | 282.4 | HR | 6.7 | 9.5 | 2.8 | 41.8 |
| KA | 2.2 | 4.8 | 2.6 | 118.2 | UK | 5.3 | 12.9 | 7.6 | 143.4 | PB | 5.6 | 8.2 | 2.6 | 46.4 |
| SK | 2.2 | 4.8 | 2.6 | 118.2 | GA | 7.5 | 11.2 | 3.7 | 49.3 | TN | 3.7 | 5.7 | 2.0 | 54.1 |
| TS | 3.4 | 5.8 | 2.4 | 70.6 | WB | 5.8 | 9.4 | 3.6 | 62.1 | BR | 4.7 | 6.6 | 1.9 | 40.4 |
| BR | 4.4 | 6.3 | 1.9 | 43.2 | BR | 2.5 | 5.4 | 2.9 | 116.0 | WB | 4.2 | 5.8 | 1.6 | 38.1 |
| HR | 6.9 | 8.8 | 1.9 | 27.5 | UP | 4.8 | 7.5 | 2.7 | 56.3 | TS | 3.0 | 4.3 | 1.3 | 43.3 |
| WB | 4.6 | 6.2 | 1.6 | 34.8 | TS | 4.3 | 6.8 | 2.5 | 58.1 | KA | 3.4 | 4.7 | 1.3 | 38.2 |
| AR | 2.4 | 3.7 | 1.3 | 54.2 | AS | 4.4 | 6.5 | 2.1 | 47.7 | MH | 4.6 | 5.8 | 1.2 | 26.1 |
| TN | 4.4 | 5.7 | 1.3 | 29.5 | SK | 4.2 | 6.3 | 2.1 | 50.0 | MP | 4.9 | 6.0 | 1.1 | 22.4 |
| MP | 4.7 | 5.6 | 0.9 | 19.1 | MZ | 4.9 | 6.7 | 1.8 | 36.7 | AP | 3.3 | 4.1 | 0.8 | 24.2 |
| MN | 9.2 | 9.8 | 0.6 | 6.5 | NL | 3.8 | 5.4 | 1.6 | 42.1 | UK | 6.6 | 7.4 | 0.8 | 12.1 |
| OD | 7.0 | 7.5 | 0.5 | 7.1 | GJ | 3.3 | 4.8 | 1.5 | 45.5 | AS | 5.4 | 6.2 | 0.8 | 14.8 |
| JH | 5.5 | 5.9 | 0.4 | 7.3 | MP | 2.6 | 3.7 | 1.1 | 42.3 | KL | 4.3 | 4.8 | 0.5 | 11.6 |
| UK | 8.0 | 8.2 | 0.2 | 2.5 | MH | 3.9 | 5.0 | 1.1 | 28.2 | OD | 7.2 | 7.7 | 0.5 | 6.9 |
| RJ | 6.9 | 7.0 | 0.1 | 1.4 | OD | 5.7 | 6.5 | 0.8 | 14.0 | JH | 5.4 | 5.7 | 0.3 | 5.6 |
| NL | 5.6 | 5.6 | 0 | 0 | MN | 8.1 | 8.7 | 0.6 | 7.4 | RJ | 6.3 | 6.0 | -0.3 | -4.8 |
| GJ | 6.2 | 6.1 | -0.1 | -1.6 | JK | 4.5 | 4.9 | 0.4 | 8.9 | AR | 4.8 | 4.4 | -0.4 | -8.3 |
| MH | 4.6 | 4.4 | -0.2 | -4.3 | KA | 4.5 | 4.6 | 0.1 | 2.2 | GJ | 5.2 | 4.6 | -0.6 | -11.5 |
| PB | 7.2 | 6.9 | -0.3 | -4.2 | ML | 3.6 | 3.5 | -0.1 | -2.8 | HP | 7.2 | 6.5 | -0.7 | -9.7 |
| UP | 8.3 | 7.9 | -0.4 | -4.8 | RJ | 6.4 | 6.1 | -0.3 | -4.7 | SK | 5.6 | 4.7 | -0.9 | -16.1 |
| CG | 6.0 | 5.1 | -0.9 | -15.0 | CG | 4.6 | 4.1 | -0.5 | -10.9 | UP | 8.5 | 7.4 | -1.1 | -12.9 |
| HP | 8.7 | 7.6 | -1.1 | -12.6 | JH | 5.2 | 4.2 | -1.0 | -19.2 | CG | 5.5 | 4.2 | -1.3 | -23.6 |
| AS | 5.5 | 4.1 | -1.4 | -25.5 | AR | 4.3 | 2.9 | -1.4 | -32.6 | MN | 13.5 | 11.9 | -1.6 | -11.9 |
| KL | 6.7 | 5.0 | -1.7 | -25.4 | HR | 7.4 | 5.7 | -1.7 | -23.0 | TR | 6.0 | 4.3 | -1.7 | -28.3 |
| TR | 6.7 | 4.8 | -1.9 | -28.4 | KL | 7.9 | 4.7 | -3.2 | -40.5 | GA | 4.0 | 2.1 | -1.9 | -47.5 |
| MZ | 3.5 | 1.6 | -1.9 | -54.3 | TR | 7.2 | 3.3 | -3.9 | -54.2 | JK | 9.4 | 7.4 | -2.0 | -21.3 |
| JK | 7.8 | 4.8 | -3.0 | -38.5 | AP | 7.8 | 3.9 | -3.9 | -50.0 | MZ | 7.2 | 3.7 | -3.5 | -48.6 |
| ML | 5.5 | 2.2 | -3.3 | -60.0 | HP | 6.1 | 2.0 | -4.1 | -67.2 |  |  |  |  |  |
| **India** | **5.7** | **7.4** | **1.7** | **29.8** | **India** | **4.5** | **5.7** | **1.2** | **26.7** | **India** | **5.6** | **7.3** | **1.7** | **30.4** |
| ** Proportion of birth outcomes of the last pregnancy in the five years preceding the survey of women age 15-49; AC-absolute changes, RC-Relative changes* | | | | | | | | | | | | | |  |

| **Additional file 3.1: Changes in proportion of stillbirth among different age groups across the States/UTs during 2015-16 and 2019-21 *** | | | | | | | | | | | | | | | | | | |  |
| --- | --- | --- | --- | --- | --- | --- | --- | --- | --- | --- | --- | --- | --- | --- | --- | --- | --- | --- | --- |
| **Age of 15-19** | | | | | **Age of 20-29** | | | | | **Age of 30-39** | | | | | **Age of 40-49** | | | | |
| States /UTs | NFHS-4 | NFHS-5 | AC | RC | States /UTs | NFHS-4 | NFHS-5 | AC | RC | States /UTs | NFHS-4 | NFHS-5 | AC | RC | States /UTs | NFHS-4 | NFHS-5 | AC | RC |
| PB | 0 | 1.3 | 1.3 | NC | ML | 0.4 | 1.0 | 0.6 | 150.0 | SK | 1.0 | 2.1 | 1.1 | 110.0 | WB | 0 | 5.2 | 5.2 | NC |
| JH | 0.8 | 1.7 | 0.9 | 112.5 | TR | 0.3 | 0.8 | 0.5 | 166.7 | UK | 0.7 | 1.5 | 0.8 | 114.3 | KA | 0 | 1.4 | 1.4 | NC |
| MP | 1.1 | 2.0 | 0.9 | 81.8 | AS | 0.3 | 0.7 | 0.4 | 133.3 | GJ | 0.6 | 1.0 | 0.4 | 66.7 | MN | 0.9 | 2.3 | 1.4 | 155.6 |
| OD | 0.4 | 1.2 | 0.8 | 200.0 | HP | 0.1 | 0.4 | 0.3 | 300.0 | JK | 0.8 | 1.0 | 0.2 | 25.0 | HR | 0.5 | 1.7 | 1.2 | 240.0 |
| KA | 0.9 | 1.7 | 0.8 | 88.9 | OD | 0.7 | 1.0 | 0.3 | 42.9 | HP | 0.1 | 0.2 | 0.1 | 100.0 | OD | 0.5 | 0.8 | 0.3 | 60.0 |
| TR | 0 | 0.6 | 0.6 | NC | WB | 0.4 | 0.6 | 0.2 | 50.0 | GA | 0 | 0 | 0 | 0 | ML | 0.7 | 1.0 | 0.3 | 42.9 |
| HP | 0 | 0 | 0 | 0 | TN | 0.3 | 0.4 | 0.1 | 33.3 | ML | 0.6 | 0.6 | 0 | 0 | NL | 1.0 | 1.3 | 0.3 | 30.0 |
| ML | 0 | 0 | 0 | 0 | MN | 0.3 | 0.4 | 0.1 | 33.3 | OD | 0.6 | 0.6 | 0 | 0 | AS | 2.0 | 2.1 | 0.1 | 5.0 |
| TN | 0 | 0 | 0 | 0 | MH | 0.3 | 0.4 | 0.1 | 33.3 | MN | 0.3 | 0.3 | 0 | 0 | HP | 0 | 0 | 0 | 0 |
| MN | 0 | 0 | 0 | 0 | TS | 0.5 | 0.6 | 0.1 | 20.0 | BR | 1.1 | 1.1 | 0 | 0 | GA | 0 | 0 | 0 | 0 |
| TS | 0 | 0 | 0 | 0 | HR | 0.7 | 0.8 | 0.1 | 14.3 | MP | 0.6 | 0.6 | 0 | 0 | KL | 0 | 0 | 0 | 0 |
| UK | 0 | 0 | 0 | 0 | BR | 0.7 | 0.8 | 0.1 | 14.3 | RJ | 0.8 | 0.8 | 0 | 0 | JH | 0.7 | 0.7 | 0 | 0 |
| HR | 0 | 0 | 0 | 0 | MP | 0.5 | 0.5 | 0 | 0 | NL | 0.6 | 0.5 | -0.1 | -16.7 | BR | 1.0 | 0.8 | -0.2 | -20.0 |
| JK | 0 | 0 | 0 | 0 | GA | 0 | 0 | 0 | 0 | JH | 1.2 | 1.1 | -0.1 | -8.3 | CG | 0.8 | 0.5 | -0.3 | -37.5 |
| AS | 0.4 | 0.4 | 0 | 0 | PB | 0.7 | 0.7 | 0 | 0 | KA | 0.7 | 0.6 | -0.1 | -14.3 | AR | 1.2 | 0.9 | -0.3 | -25.0 |
| MH | 0.6 | 0.3 | -0.3 | -50.0 | RJ | 0.6 | 0.5 | -0.1 | -16.7 | WB | 0.8 | 0.6 | -0.2 | -25.0 | RJ | 0.6 | 0.3 | -0.3 | -50.0 |
| GJ | 0.5 | 0.2 | -0.3 | -60.0 | JK | 0.5 | 0.4 | -0.1 | -20.0 | MZ | 0.4 | 0.2 | -0.2 | -50.0 | PB | 0.4 | 0 | -0.4 | -100.0 |
| UP | 1.9 | 1.5 | -0.4 | -21.1 | NL | 0.2 | 0.1 | -0.1 | -50.0 | MH | 0.6 | 0.4 | -0.2 | -33.3 | GJ | 1.8 | 0.9 | -0.9 | -50.0 |
| RJ | 1.2 | 0.8 | -0.4 | -33.3 | GJ | 0.4 | 0.3 | -0.1 | -25.0 | TS | 0.6 | 0.4 | -0.2 | -33.3 | UK | 1.3 | 0.4 | -0.9 | -69.2 |
| AR | 0.4 | 0 | -0.4 | -100.0 | KA | 0.4 | 0.2 | -0.2 | -50.0 | KL | 0.4 | 0.2 | -0.2 | -50.0 | UP | 1.9 | 0.9 | -1.0 | -52.6 |
| CG | 1.3 | 0.8 | -0.5 | -38.5 | AP | 0.4 | 0.2 | -0.2 | -50.0 | TR | 1.1 | 0.8 | -0.3 | -27.3 | MP | 1.3 | 0 | -1.3 | -100.0 |
| WB | 1.1 | 0.6 | -0.5 | -45.5 | MZ | 0.3 | 0.1 | -0.2 | -66.7 | AS | 0.7 | 0.4 | -0.3 | -42.9 | TN | 1.4 | 0 | -1.4 | -100.0 |
| BR | 2.6 | 1.7 | -0.9 | -34.6 | KL | 0.2 | 0 | -0.2 | -100.0 | PB | 0.7 | 0.4 | -0.3 | -42.9 | MZ | 2.6 | 0 | -2.6 | -100.0 |
| NL | 1.2 | 0 | -1.2 | -100.0 | CG | 0.9 | 0.6 | -0.3 | -33.3 | AR | 0.8 | 0.5 | -0.3 | -37.5 | TR | 2.8 | 0 | -2.8 | -100.0 |
| AP | 2.8 | 1.5 | -1.3 | -46.4 | JH | 1.0 | 0.7 | -0.3 | -30.0 | TN | 0.5 | 0.2 | -0.3 | -60.0 | MH | 3.2 | 0 | -3.2 | -100.0 |
| MZ | 1.7 | 0 | -1.7 | -100.0 | UP | 1.3 | 1.0 | -0.3 | -23.1 | UP | 1.4 | 1.0 | -0.4 | -28.6 | JK | 3.7 | 0.3 | -3.4 | -91.9 |
|  |  |  |  |  | UK | 0.9 | 0.6 | -0.3 | -33.3 | CG | 1.4 | 0.9 | -0.5 | -35.7 |  |  |  |  |  |
|  |  |  |  |  | AR | 0.5 | 0.2 | -0.3 | -60.0 | AP | 1.1 | 0.5 | -0.6 | -54.5 |  |  |  |  |  |
|  |  |  |  |  | SK | 0.7 | 0.0 | -0.7 | -100.0 | HR | 1.3 | 0.5 | -0.8 | -61.5 |  |  |  |  |  |
| ** Proportion of birth outcomes of the last pregnancy in the five years preceding the survey of women age 15-49; AC-absolute changes, RC-Relative changes; NC-Not calculated* | | | | | | | | | | | | | | | | | | | |

| **Additional file 3.2: Changes in proportion of stillbirth in different settings across the States/UTs during 2015-16 and 2019-21 *** | | | | | | | | | |
| --- | --- | --- | --- | --- | --- | --- | --- | --- | --- |
| **Urban** | | | | | **Rural** | | | | |
| States /UTs | NFHS-4 | NFHS-5 | AC | RC | States /UTs | NFHS-4 | NFHS-5 | AC | RC |
| SK | 0.8 | 2.7 | 1.9 | 237.5 | UK | 0.9 | 1.3 | 0.4 | 44.4 |
| HP | 0 | 1.0 | 1.0 | NC | ML | 0.4 | 0.7 | 0.3 | 75.0 |
| ML | 0.7 | 1.5 | 0.8 | 114.3 | OD | 0.7 | 0.9 | 0.2 | 28.6 |
| TR | 0.8 | 1.5 | 0.7 | 87.5 | TR | 0.4 | 0.6 | 0.2 | 50.0 |
| WB | 0.3 | 0.8 | 0.5 | 166.7 | MN | 0.3 | 0.5 | 0.2 | 66.7 |
| JH | 0.6 | 1.1 | 0.5 | 83.3 | HP | 0.1 | 0.2 | 0.1 | 100.0 |
| BR | 0.9 | 1.4 | 0.5 | 55.6 | TN | 0.3 | 0.4 | 0.1 | 33.3 |
| TS | 0.2 | 0.6 | 0.4 | 200.0 | AS | 0.6 | 0.7 | 0.1 | 16.7 |
| JK | 0.5 | 0.8 | 0.3 | 60.0 | WB | 0.6 | 0.6 | 0 | 0 |
| AS | 0.1 | 0.3 | 0.2 | 200.0 | GA | 0 | 0 | 0 | 0 |
| MN | 0.4 | 0.5 | 0.1 | 25.0 | BR | 0.9 | 0.9 | 0 | 0 |
| OD | 0.4 | 0.5 | 0.1 | 25.0 | GJ | 0.5 | 0.5 | 0 | 0 |
| MP | 0.5 | 0.6 | 0.1 | 20.0 | KA | 0.5 | 0.5 | 0 | 0 |
| GA | 0 | 0 | 0 | 0 | HR | 0.9 | 0.8 | -0.1 | -11.1 |
| AR | 0.3 | 0.3 | 0 | 0 | PB | 0.8 | 0.7 | -0.1 | -12.5 |
| GJ | 0.5 | 0.5 | 0 | 0 | TS | 0.7 | 0.6 | -0.1 | -14.3 |
| RJ | 0.6 | 0.6 | 0 | 0 | RJ | 0.7 | 0.6 | -0.1 | -14.3 |
| UP | 1.1 | 1.0 | -0.1 | -9.1 | MP | 0.6 | 0.5 | -0.1 | -16.7 |
| HR | 0.6 | 0.5 | -0.1 | -16.7 | NL | 0.5 | 0.4 | -0.1 | -20.0 |
| MH | 0.5 | 0.4 | -0.1 | -20.0 | MH | 0.4 | 0.3 | -0.1 | -25.0 |
| PB | 0.5 | 0.4 | -0.1 | -20.0 | KL | 0.3 | 0.2 | -0.1 | -33.3 |
| TN | 0.4 | 0.3 | -0.1 | -25.0 | AP | 0.6 | 0.4 | -0.2 | -33.3 |
| NL | 0.3 | 0.2 | -0.1 | -33.3 | JH | 1.1 | 0.8 | -0.3 | -27.3 |
| KL | 0.2 | 0 | -0.2 | -100.0 | CG | 1.0 | 0.7 | -0.3 | -30.0 |
| KA | 0.5 | 0.2 | -0.3 | -60.0 | MZ | 0.5 | 0.2 | -0.3 | -60.0 |
| AP | 0.8 | 0.3 | -0.5 | -62.5 | JK | 0.9 | 0.6 | -0.3 | -33.3 |
| CG | 1.3 | 0.7 | -0.6 | -46.2 | AR | 0.7 | 0.4 | -0.3 | -42.9 |
| MZ | 0.6 | 0 | -0.6 | -100.0 | UP | 1.4 | 1 | -0.4 | -28.6 |
| UK | 0.8 | 0.1 | -0.7 | -87.5 | SK | 0.8 | 0 | -0.8 | -100.0 |
| **India** | **0.6** | **0.7** | **0.1** | **16.7** | **India** | **0.8** | **0.9** | **0.1** | **12.5** |
| ** Proportion of birth outcomes of the last pregnancy in the five years preceding the survey of women age 15-49; AC-absolute changes, RC-Relative changes; NC-Not calculated* | | | | | | | | | |

| **Additional file 3.3: Changes in proportion of stillbirth in different community levels across the States/UTs during 2015-16 and 2019-21 *** | | | | | | | | | | | | | | | |
| --- | --- | --- | --- | --- | --- | --- | --- | --- | --- | --- | --- | --- | --- | --- | --- |
| **SC** | | | | | **ST** | | | | | **OBC** | | | | | |
| States /UTs | NFHS-4 | NFHS-5 | AC | RC | States /UTs | NFHS-4 | NFHS-5 | AC | RC | States /UTs | NFHS-4 | NFHS-5 | AC | RC |  |
| MZ | 0 | 1.6 | 1.6 | 100.0 | UK | 0 | 3.2 | 3.2 | NC | TR | 0.0 | 1.4 | 1.4 | NC |  |
| HP | 0 | 0.3 | 0.3 | 100.0 | HR | 0 | 2.6 | 2.6 | NC | SK | 1.0 | 1.9 | 0.9 | 90.0 |  |
| AS | 0.3 | 0.5 | 0.2 | 66.7 | WB | 0 | 2.4 | 2.4 | NC | MN | 0.1 | 0.9 | 0.8 | 800.0 |  |
| TS | 0.7 | 0.9 | 0.2 | 28.6 | HP | 0.2 | 2.2 | 2.0 | 1000.0 | AS | 0.5 | 1.0 | 0.5 | 100.0 |  |
| BR | 0.9 | 1.1 | 0.2 | 22.2 | TN | 0 | 0.8 | 0.8 | NC | OD | 0.7 | 1.2 | 0.5 | 71.4 |  |
| KL | 0.5 | 0.6 | 0.1 | 20.0 | TR | 0.2 | 0.7 | 0.5 | 250.0 | TS | 0.1 | 0.5 | 0.4 | 400.0 |  |
| ML | 0 | 0 | 0 | 0 | BR | 0.7 | 1.2 | 0.5 | 71.4 | PB | 0.1 | 0.5 | 0.4 | 400.0 |  |
| SK | 0 | 0 | 0 | 0 | GJ | 0.1 | 0.4 | 0.3 | 300.0 | HP | 0 | 0.2 | 0.2 | NC |  |
| WB | 0.5 | 0.5 | 0 | 0 | ML | 0.4 | 0.7 | 0.3 | 75.0 | WB | 0.3 | 0.5 | 0.2 | 66.7 |  |
| TN | 0.3 | 0.3 | 0 | 0 | AS | 0.3 | 0.5 | 0.2 | 66.7 | JK | 0.4 | 0.6 | 0.2 | 50.0 |  |
| OD | 0.8 | 0.7 | -0.1 | -12.5 | RJ | 0.6 | 0.8 | 0.2 | 33.3 | BR | 0.8 | 0.9 | 0.1 | 12.5 |  |
| RJ | 0.8 | 0.7 | -0.1 | -12.5 | UP | 1.1 | 1.3 | 0.2 | 18.2 | GA | 0 | 0 | 0 | 0 |  |
| MP | 0.6 | 0.5 | -0.1 | -16.7 | GA | 0 | 0 | 0 | 0 | MZ | 0 | 0 | 0 | 0 |  |
| JK | 0.2 | 0 | -0.2 | -100.0 | KL | 0 | 0 | 0 | 0 | TN | 0.4 | 0.4 | 0 | 0 |  |
| UP | 1.5 | 1.2 | -0.3 | -20.0 | OD | 0.8 | 0.8 | 0 | 0 | MP | 0.6 | 0.5 | -0.1 | -16.7 |  |
| PB | 1.1 | 0.8 | -0.3 | -27.3 | JH | 1.1 | 1.1 | 0 | 0 | AP | 0.6 | 0.5 | -0.1 | -16.7 |  |
| JH | 1.0 | 0.7 | -0.3 | -30.0 | CG | 0.9 | 0.9 | 0 | 0 | GJ | 0.5 | 0.4 | -0.1 | -20.0 |  |
| AP | 0.8 | 0.5 | -0.3 | -37.5 | KA | 0.6 | 0.6 | 0 | 0 | KA | 0.4 | 0.3 | -0.1 | -25.0 |  |
| GJ | 0.6 | 0.3 | -0.3 | -50.0 | MN | 0.5 | 0.5 | 0 | 0 | KL | 0.2 | 0.1 | -0.1 | -50.0 |  |
| MH | 0.3 | 0.0 | -0.3 | -100.0 | NL | 0.5 | 0.4 | -0.1 | -20.0 | HR | 0.6 | 0.4 | -0.2 | -33.3 |  |
| HR | 1.2 | 0.8 | -0.4 | -33.3 | MP | 0.7 | 0.5 | -0.2 | -28.6 | MH | 0.5 | 0.3 | -0.2 | -40.0 |  |
| KA | 0.8 | 0.4 | -0.4 | -50.0 | AR | 0.6 | 0.4 | -0.2 | -33.3 | UK | 1.8 | 1.5 | -0.3 | -16.7 |  |
| UK | 0.8 | 0.4 | -0.4 | -50.0 | MH | 0.6 | 0.3 | -0.3 | -50.0 | UP | 1.3 | 1.0 | -0.3 | -23.1 |  |
| NL | 0.5 | 0.0 | -0.5 | -100.0 | MZ | 0.6 | 0.1 | -0.5 | -83.3 | RJ | 0.7 | 0.4 | -0.3 | -42.9 |  |
| AR | 1.0 | 0.3 | -0.7 | -70.0 | AP | 0.7 | 0 | -0.7 | -100.0 | JH | 1.1 | 0.7 | -0.4 | -36.4 |  |
| MN | 0.7 | 0 | -0.7 | -100.0 | SK | 0.9 | 0 | -0.9 | -100.0 | CG | 1.1 | 0.6 | -0.5 | -45.5 |  |
| TR | 1.7 | 0.9 | -0.8 | -47.1 | JK | 1.5 | 0.3 | -1.2 | -80.0 | AR | 1.9 | 0.2 | -1.7 | -89.5 |  |
| CG | 1.4 | 0.4 | -1.0 | -71.4 | TS | 1.9 | 0.1 | -1.8 | -94.7 |  |  |  |  |  |  |
| **India** | **0.9** | **1.0** | **0.1** | **11.1** | **India** | **0.7** | **0.8** | **0.1** | **14.3** | **India** | **0.8** | **0.8** | **0** | **0** |  |
| ** Proportion of birth outcomes of the last pregnancy in the five years preceding the survey of women age 15-49; AC-absolute changes, RC-Relative changes; NC-Not calculated* | | | | | | | | | | | | | | | |

| **Additional file 4.1: Changes in proportion of abortion among different age groups across the States/UTs during 2015-16 and 2019-21 *** | | | | | | | | | | | | | | | | | | | | |
| --- | --- | --- | --- | --- | --- | --- | --- | --- | --- | --- | --- | --- | --- | --- | --- | --- | --- | --- | --- | --- |
| **Age of 15-19** | | | | | **Age of 20-29** | | | | | **Age of 30-39** | | | | | **Age of 40-49** | | | | | |
| States /UTs | NFHS-4 | NFHS-5 | AC | RC | States /UTs | NFHS-4 | NFHS-5 | AC | RC | States /UTs | NFHS-4 | NFHS-5 | AC | RC | States /UTs | NFHS-4 | NFHS-5 | AC | RC |  |
| TS | 0.7 | 8.0 | 7.3 | 1042.9 | GA | 1.2 | 5.5 | 4.3 | 358.3 | MN | 12.5 | 14.8 | 2.3 | 18.4 | GA | 0.0 | 14.7 | 14.7 | NC |  |
| PB | 2.4 | 7.3 | 4.9 | 204.2 | TR | 3.4 | 6.8 | 3.4 | 100.0 | BR | 1.7 | 3.6 | 1.9 | 111.8 | MH | 1.4 | 8.5 | 7.1 | 507.1 |  |
| GJ | 2.4 | 6.1 | 3.7 | 154.2 | SK | 1.0 | 2.1 | 1.1 | 110.0 | TR | 8.3 | 9.2 | 0.9 | 10.8 | UK | 7.1 | 14.0 | 6.9 | 97.2 |  |
| UK | 1.5 | 4.3 | 2.8 | 186.7 | AP | 2.5 | 3.3 | 0.8 | 32.0 | MP | 2.2 | 3.0 | 0.8 | 36.4 | HR | 1.2 | 7.9 | 6.7 | 558.3 |  |
| KA | 0.3 | 3.0 | 2.7 | 900.0 | HR | 1.5 | 2.2 | 0.7 | 46.7 | HR | 3.3 | 4.1 | 0.8 | 24.2 | BR | 2.5 | 6.3 | 3.8 | 152.0 |  |
| TR | 3.5 | 6.2 | 2.7 | 77.1 | BR | 1.1 | 1.7 | 0.6 | 54.5 | TN | 4.9 | 5.7 | 0.8 | 16.3 | MN | 19.0 | 22.6 | 3.6 | 18.9 |  |
| HR | 1.8 | 2.7 | 0.9 | 50.0 | TS | 2.8 | 3.4 | 0.6 | 21.4 | MH | 5.1 | 5.8 | 0.7 | 13.7 | MP | 3.9 | 5.4 | 1.5 | 38.5 |  |
| JH | 1.2 | 1.4 | 0.2 | 16.7 | OD | 3.7 | 4.3 | 0.6 | 16.2 | KA | 2.3 | 2.8 | 0.5 | 21.7 | HP | 7.1 | 8.0 | 0.9 | 12.7 |  |
| MH | 2.9 | 2.9 | 0 | 0 | JH | 2.2 | 2.6 | 0.4 | 18.2 | NL | 2.2 | 2.5 | 0.3 | 13.6 | RJ | 2.6 | 3.4 | 0.8 | 30.8 |  |
| AS | 2.9 | 2.9 | 0 | 0 | HP | 2.3 | 2.7 | 0.4 | 17.4 | MZ | 0.2 | 0.4 | 0.2 | 100.0 | TN | 10.5 | 11.1 | 0.6 | 5.7 |  |
| UP | 4.0 | 3.5 | -0.5 | -12.5 | KA | 1.6 | 1.9 | 0.3 | 18.8 | PB | 3.4 | 3.4 | 0 | 0 | OD | 8.7 | 8.9 | 0.2 | 2.3 |  |
| AP | 3.4 | 2.8 | -0.6 | -17.6 | UK | 2.6 | 2.9 | 0.3 | 11.5 | GJ | 3.0 | 2.9 | -0.1 | -3.3 | AS | 10.9 | 10.8 | -0.1 | -0.9 |  |
| BR | 1.1 | 0.4 | -0.7 | -63.6 | PB | 2.2 | 2.4 | 0.2 | 9.1 | SK | 1.5 | 1.4 | -0.1 | -6.7 | CG | 3.2 | 3.0 | -0.2 | -6.3 |  |
| NL | 5.3 | 4.5 | -0.8 | -15.1 | TN | 3.0 | 3.1 | 0.1 | 3.3 | UK | 4.8 | 4.5 | -0.3 | -6.3 | JK | 4.3 | 4.0 | -0.3 | -7.0 |  |
| AR | 5.1 | 4.3 | -0.8 | -15.7 | MN | 7.6 | 7.7 | 0.1 | 1.3 | CG | 2.7 | 2.3 | -0.4 | -14.8 | MZ | 0.5 | 0.0 | -0.5 | -100 |  |
| RJ | 3.2 | 2.4 | -0.8 | -25.0 | MZ | 0.1 | 0.1 | 0 | 0 | HP | 2.9 | 2.5 | -0.4 | -13.8 | JH | 3.8 | 3.0 | -0.8 | -21.1 |  |
| OD | 3.1 | 2.0 | -1.1 | -35.5 | NL | 1.7 | 1.7 | 0 | 0 | AP | 4.4 | 3.9 | -0.5 | -11.4 | GJ | 5.9 | 4.7 | -1.2 | -20.3 |  |
| MP | 1.9 | 0.6 | -1.3 | -68.4 | GJ | 1.7 | 1.5 | -0.2 | -11.8 | AS | 7.3 | 6.5 | -0.8 | -11.0 | ML | 1.6 | 0.3 | -1.3 | -81.3 |  |
| ML | 2.1 | 0.3 | -1.8 | -85.7 | RJ | 1.5 | 1.3 | -0.2 | -13.3 | OD | 6.7 | 5.8 | -0.9 | -13.4 | UP | 8.9 | 7.4 | -1.5 | -16.9 |  |
| WB | 3.7 | 1.4 | -2.3 | -62.2 | MP | 1.3 | 1.1 | -0.2 | -15.4 | JH | 3.9 | 2.9 | -1.0 | -25.6 | NL | 2.4 | 0.8 | -1.6 | -66.7 |  |
| TN | 4.1 | 1.5 | -2.6 | -63.4 | MH | 3.5 | 3.2 | -0.3 | -8.6 | ML | 1.3 | 0.2 | -1.1 | -84.6 | AR | 3.1 | 1.1 | -2.0 | -64.5 |  |
| JK | 3.6 | 0 | -3.6 | -100.0 | AS | 4.6 | 4.3 | -0.3 | -6.5 | RJ | 3.2 | 2.0 | -1.2 | -37.5 | PB | 7.9 | 4.4 | -3.5 | -44.3 |  |
| MN | 6.1 | 1.3 | -4.8 | -78.7 | CG | 2.2 | 1.7 | -0.5 | -22.7 | AR | 4.1 | 2.7 | -1.4 | -34.1 | KL | 9.6 | 5.8 | -3.8 | -39.6 |  |
| CG | 7.4 | 1.1 | -6.3 | -85.1 | UP | 3.6 | 2.8 | -0.8 | -22.2 | JK | 4.2 | 2.7 | -1.5 | -35.7 | KA | 13.7 | 4.2 | -9.5 | -69.3 |  |
|  |  |  |  |  | ML | 1.0 | 0.1 | -0.9 | -90 | WB | 9.2 | 7.4 | -1.8 | -19.6 | TR | 23.4 | 13.7 | -9.7 | -41.5 |  |
|  |  |  |  |  | WB | 4.2 | 3.0 | -1.2 | -28.6 | KL | 4.6 | 2.6 | -2.0 | -43.5 | WB | 21.4 | 5.4 | -16.0 | -74.8 |  |
|  |  |  |  |  | AR | 3.9 | 2.7 | -1.2 | -30.8 | UP | 7.5 | 5.2 | -2.3 | -30.7 |  |  |  |  |  |  |
|  |  |  |  |  | JK | 2.9 | 1.5 | -1.4 | -48.3 | TS | 6.4 | 3.7 | -2.7 | -42.2 |  |  |  |  |  |  |
|  |  |  |  |  | KL | 4.4 | 2.6 | -1.8 | -40.9 | GA | 5.8 | 1.9 | -3.9 | -67.2 |  |  |  |  |  |  |
| ** Proportion of birth outcomes of the last pregnancy in the five years preceding the survey of women age 15-49; AC-absolute changes, RC-Relative changes* | | | | | | | | | | | | | | | | | | |  | |

| **Additional file 4.2: Changes in proportion of abortion in different settings across the States/UTs during 2015-16 and 2019-21 *** | | | | | | | | | |
| --- | --- | --- | --- | --- | --- | --- | --- | --- | --- |
| **Urban** | | | | | **Rural** | | | | |
| States /UTs | NFHS-4 | NFHS-5 | AC | RC | States /UTs | NFHS-4 | NFHS-5 | AC | RC |
| TR | 6.0 | 10.8 | 4.8 | 80.0 | GA | 1.8 | 5.0 | 3.2 | 177.8 |
| SK | 0.0 | 4.5 | 4.5 | NC | TR | 4.8 | 6.5 | 1.7 | 35.4 |
| HP | 0.4 | 3.2 | 2.8 | 700.0 | TS | 2.0 | 3.4 | 1.4 | 70.0 |
| MN | 11.8 | 13.7 | 1.9 | 16.1 | MN | 10.2 | 11.4 | 1.2 | 11.8 |
| OD | 5.3 | 7.1 | 1.8 | 34.0 | BR | 1.2 | 2.2 | 1.0 | 83.3 |
| TN | 3.6 | 5.1 | 1.5 | 41.7 | HR | 1.7 | 2.7 | 1.0 | 58.8 |
| HR | 2.2 | 3.5 | 1.3 | 59.1 | KA | 1.5 | 2.1 | 0.6 | 40.0 |
| AP | 3.5 | 4.2 | 0.7 | 20.0 | JH | 2.2 | 2.6 | 0.4 | 18.2 |
| GA | 4.2 | 4.7 | 0.5 | 11.9 | AP | 2.6 | 3.0 | 0.4 | 15.4 |
| BR | 1.9 | 2.3 | 0.4 | 21.1 | PB | 2.4 | 2.8 | 0.4 | 16.7 |
| UK | 3.7 | 4.1 | 0.4 | 10.8 | MH | 2.5 | 2.8 | 0.3 | 12.0 |
| MZ | 0.2 | 0.3 | 0.1 | 50.0 | UK | 3.1 | 3.4 | 0.3 | 9.7 |
| PB | 3.1 | 3.2 | 0.1 | 3.2 | NL | 1.3 | 1.5 | 0.2 | 15.4 |
| MH | 5.3 | 5.4 | 0.1 | 1.9 | MP | 1.0 | 1.1 | 0.1 | 10.0 |
| MP | 2.9 | 2.9 | 0 | 0 | HP | 2.7 | 2.7 | 0 | 0 |
| GJ | 3.2 | 3.2 | 0 | 0 | CG | 1.9 | 1.8 | -0.1 | -5.3 |
| KA | 2.3 | 2.2 | -0.1 | -4.3 | GJ | 1.4 | 1.3 | -0.1 | -7.1 |
| NL | 3.7 | 3.5 | -0.2 | -5.4 | MZ | 0.2 | 0.1 | -0.1 | -50.0 |
| TS | 4.8 | 4.1 | -0.7 | -14.6 | AS | 5.1 | 4.9 | -0.2 | -3.9 |
| RJ | 3.3 | 2.4 | -0.9 | -27.3 | OD | 4.6 | 4.3 | -0.3 | -6.5 |
| ML | 1.9 | 0.5 | -1.4 | -73.7 | RJ | 1.6 | 1.2 | -0.4 | -25.0 |
| JH | 4.1 | 2.6 | -1.5 | -36.6 | TN | 3.6 | 3.1 | -0.5 | -13.9 |
| AR | 4.6 | 2.8 | -1.8 | -39.1 | JK | 3.1 | 2.2 | -0.9 | -29.0 |
| AS | 8.1 | 6.2 | -1.9 | -23.5 | WB | 4.4 | 3.4 | -1.0 | -22.7 |
| CG | 4.3 | 2.2 | -2.1 | -48.8 | ML | 1.1 | 0.1 | -1.0 | -90.9 |
| KL | 5.3 | 3.1 | -2.2 | -41.5 | UP | 4.6 | 3.5 | -1.1 | -23.9 |
| UP | 6.9 | 4.6 | -2.3 | -33.3 | SK | 1.7 | 0.6 | -1.1 | -64.7 |
| JK | 4.8 | 2.1 | -2.7 | -56.3 | AR | 3.8 | 2.7 | -1.1 | -28.9 |
| WB | 7.7 | 4.7 | -3.0 | -39.0 | KL | 3.9 | 2.4 | -1.5 | -38.5 |
| **India** | **4.7** | **4.0** | **-0.7** | **-14.9** | **India** | **2.9** | **2.5** | **-0.4** | **-13.8** |
| ** Proportion of birth outcomes of the last pregnancy in the five years preceding the survey of women age 15-49; AC-absolute changes, RC-Relative changes* | | | | | | | | | |

| **Additional file 5: Trends in proportion of place of abortion among Indian women across the States/UTs during 2015-16 and 2019-21 *** | | | | | | | | | | | | | | |
| --- | --- | --- | --- | --- | --- | --- | --- | --- | --- | --- | --- | --- | --- | --- |
| **Public health sector** | | | | | **Private health sector** | | | | | **At home** | | | | |
| **States**  **/UTs** | **NFHS-4** | **NFHS-5** | **AC** | **RC** | **States /UTs** | **NFHS-4** | **NFHS-5** | **AC** | **RC** | **States /UTs** | **NFHS-4** | **NFHS-5** | **AC** | **RC** |
| KL | 20.9 | 48.5 | 27.6 | 132.1 | HP | 27.0 | 39.4 | 12.4 | 45.9 | PB | 13.0 | 34.0 | 21.0 | 161.5 |
| JK | 54.3 | 79.4 | 25.1 | 46.2 | TS | 69.6 | 79.1 | 9.5 | 13.6 | RJ | 18.7 | 38.6 | 19.9 | 106.4 |
| NL | 14.4 | 32.0 | 17.6 | 122.2 | UK | 38.4 | 47.3 | 8.9 | 23.2 | TR | 26.3 | 45.2 | 18.9 | 71.9 |
| WB | 13.8 | 23.9 | 10.1 | 73.2 | UP | 44.3 | 52.3 | 8.0 | 18.1 | BR | 22.4 | 39.8 | 17.4 | 77.7 |
| PB | 14.3 | 20.6 | 6.3 | 44.1 | JH | 47.7 | 54.9 | 7.2 | 15.1 | MN | 10.3 | 24.9 | 14.6 | 141.7 |
| GJ | 9.1 | 14.5 | 5.4 | 59.3 | AS | 20.3 | 26.4 | 6.1 | 30.0 | OD | 47.0 | 55.7 | 8.7 | 18.5 |
| KA | 21.6 | 26.1 | 4.5 | 20.8 | HR | 56.9 | 62.1 | 5.2 | 9.1 | HR | 21.4 | 25.4 | 4.0 | 18.7 |
| CG | 20.0 | 23.8 | 3.8 | 19.0 | NL | 54.7 | 59.4 | 4.7 | 8.6 | AR | 30.1 | 33.9 | 3.8 | 12.6 |
| AP | 16.7 | 20.0 | 3.3 | 19.8 | GJ | 65.3 | 69.2 | 3.9 | 6.0 | MH | 4.8 | 8.3 | 3.5 | 72.9 |
| BR | 9.2 | 11.9 | 2.7 | 29.3 | MP | 49.5 | 53.2 | 3.7 | 7.5 | MP | 28.9 | 31.0 | 2.1 | 7.3 |
| RJ | 21.4 | 23.9 | 2.5 | 11.7 | WB | 50.0 | 52.0 | 2.0 | 4.0 | TN | 6.7 | 8.6 | 1.9 | 28.4 |
| AR | 38.2 | 39.5 | 1.3 | 3.4 | TN | 63.9 | 64.7 | 0.8 | 1.3 | AP | 5.6 | 6.3 | 0.7 | 12.5 |
| UP | 13.9 | 13.5 | -0.4 | -2.9 | MH | 75.5 | 76.1 | 0.6 | 0.8 | KA | 13.0 | 13.4 | 0.4 | 3.1 |
| AS | 48.9 | 47.9 | -1.0 | -2.0 | CG | 35.4 | 33.4 | -2.0 | -5.6 | KL | 1.2 | 1.5 | 0.3 | 25.0 |
| UK | 17.9 | 16.6 | -1.3 | -7.3 | OD | 21.9 | 19.2 | -2.7 | -12.3 | JK | 7.3 | 6.5 | -0.8 | -11.0 |
| JH | 11.0 | 8.9 | -2.1 | -19.1 | TR | 17.1 | 14.1 | -3.0 | -17.5 | CG | 44.5 | 42.8 | -1.7 | -3.8 |
| HP | 26.8 | 24.6 | -2.2 | -8.2 | AP | 77.7 | 73.7 | -4.0 | -5.1 | JH | 40.1 | 36.2 | -3.9 | -9.7 |
| TN | 29.3 | 26.1 | -3.2 | -10.9 | KA | 65.4 | 60.2 | -5.2 | -8.0 | AS | 30.8 | 25.3 | -5.5 | -17.9 |
| MH | 19.6 | 15.6 | -4.0 | -20.4 | AR | 31.7 | 25.6 | -6.1 | -19.2 | TS | 10.9 | 5.2 | -5.7 | -52.3 |
| TS | 19.5 | 14.8 | -4.7 | -24.1 | MN | 49.4 | 41.0 | -8.4 | -17.0 | UK | 42.4 | 36.1 | -6.3 | -14.9 |
| MP | 21.1 | 15.8 | -5.3 | -25.1 | BR | 67.9 | 47.3 | -20.6 | -30.3 | UP | 41.2 | 33.9 | -7.3 | -17.7 |
| MN | 38.5 | 33.1 | -5.4 | -14.0 | RJ | 59.4 | 37.5 | -21.9 | -36.9 | GJ | 24.1 | 16.2 | -7.9 | -32.8 |
| OD | 30.2 | 24.2 | -6.0 | -19.9 | JK | 38.3 | 14.0 | -24.3 | -63.4 | HP | 46.3 | 36.0 | -10.3 | -22.2 |
| HR | 21.8 | 12.1 | -9.7 | -44.5 | PB | 72.7 | 45.4 | -27.3 | -37.6 | WB | 36.2 | 21.5 | -14.7 | -40.6 |
| TR | 56.6 | 40.4 | -16.2 | -28.6 | KL | 77.9 | 50.1 | -27.8 | -35.7 | NL | 30.0 | 6.1 | -23.9 | -79.7 |
| **India** | **20.2** | **20.3** | **0.1** | **0.5** | **India** | **52.4** | **52.9** | **0.5** | **1.0** | **India** | **27.0** | **26.2** | **-0.8** | **-3.0** |
| ** Proportion of birth outcomes of the last pregnancy in the five years preceding the survey of women age 15-49; AC-absolute changes, RC-Relative changes* | | | | | | | | | | | | | |  |

| **Additional file 6: Trends in proportion of abortion performed by among the pregnant women across the States/UTs during 2015-16 and 2019-21 *** | | | | | | | | | | | | | | | | |
| --- | --- | --- | --- | --- | --- | --- | --- | --- | --- | --- | --- | --- | --- | --- | --- | --- |
| **Doctors** | | | | | **Nurse/ANM/LHV** | | | | | | **Self** | | | | | |
| States  /UTs | NFHS-4 | NFHS-5 | AC | RC | States /UTs | NFHS-4 | NFHS-5 | AC | RC | States /UTs | | NFHS-4 | NFHS-5 | AC | RC |  |
| NL | 62.2 | 77.1 | 14.9 | 24.0 | KA | 5.6 | 11.3 | 5.7 | 101.8 | BR | | 17.0 | 41.9 | 24.9 | 146.5 |  |
| JK | 72.9 | 81.9 | 9.0 | 12.3 | MP | 19.8 | 25.0 | 5.2 | 26.3 | PB | | 11.2 | 35.8 | 24.6 | 219.6 |  |
| WB | 60.7 | 69.5 | 8.8 | 14.5 | NL | 1.4 | 5.1 | 3.7 | 264.3 | TR | | 27 | 45.8 | 18.8 | 69.6 |  |
| HR | 43.4 | 51.8 | 8.4 | 19.4 | HP | 5.3 | 7.9 | 2.6 | 49.1 | MN | | 9.0 | 23.5 | 14.5 | 161.1 |  |
| UP | 23.2 | 30.1 | 6.9 | 29.7 | KL | 0.5 | 1.2 | 0.7 | 140.0 | RJ | | 23.6 | 37.9 | 14.3 | 60.6 |  |
| GJ | 67.7 | 74.6 | 6.9 | 10.2 | GJ | 4.5 | 4.5 | 0 | 0 | AR | | 30.6 | 44.7 | 14.1 | 46.1 |  |
| UK | 37.8 | 42.8 | 5.0 | 13.2 | OD | 3.5 | 3.3 | -0.2 | -5.7 | CG | | 33.3 | 43.6 | 10.3 | 30.9 |  |
| TN | 76.1 | 80.0 | 3.9 | 5.1 | AR | 3.5 | 2.8 | -0.7 | -20.0 | JH | | 32.0 | 39.6 | 7.6 | 23.8 |  |
| AS | 60.4 | 64.1 | 3.7 | 6.1 | TS | 2.4 | 1.6 | -0.8 | -33.3 | KL | | 1.2 | 7.8 | 6.6 | 550.0 |  |
| JH | 31.3 | 34.1 | 2.8 | 8.9 | CG | 9.9 | 8.9 | -1.0 | -10.1 | OD | | 49.8 | 54.1 | 4.3 | 8.6 |  |
| HP | 53.5 | 55.9 | 2.4 | 4.5 | UK | 18.9 | 17.3 | -1.6 | -8.5 | TN | | 9.7 | 13.3 | 3.6 | 37.1 |  |
| MH | 87.9 | 89.9 | 2.0 | 2.3 | AS | 8.5 | 6.9 | -1.6 | -18.8 | AP | | 5.5 | 9.0 | 3.5 | 63.6 |  |
| TS | 88.3 | 88.8 | 0.5 | 0.6 | RJ | 23.1 | 21.4 | -1.7 | -7.4 | HR | | 18.3 | 21.4 | 3.1 | 16.9 |  |
| AP | 88.1 | 87.1 | -1.0 | -1.1 | UP | 31.3 | 29.5 | -1.8 | -5.8 | MH | | 2.5 | 5.1 | 2.6 | 104.0 |  |
| MP | 43.7 | 40.3 | -3.4 | -7.8 | AP | 4.7 | 2.1 | -2.6 | -55.3 | AS | | 27.8 | 27.1 | -0.7 | -2.5 |  |
| KA | 81.1 | 76.9 | -4.2 | -5.2 | WB | 4.4 | 0.6 | -3.8 | -86.4 | HP | | 37.3 | 36.2 | -1.1 | -2.9 |  |
| MN | 72.5 | 65.9 | -6.6 | -9.1 | MH | 7.4 | 2.8 | -4.6 | -62.2 | JK | | 7.3 | 5.7 | -1.6 | -21.9 |  |
| KL | 98.3 | 90.3 | -8.0 | -8.1 | JK | 16.0 | 10.8 | -5.2 | -32.5 | KA | | 12.1 | 9.9 | -2.2 | -18.2 |  |
| OD | 41.3 | 32.9 | -8.4 | -20.3 | BR | 30.3 | 24.0 | -6.3 | -20.8 | TS | | 7.3 | 4.8 | -2.5 | -34.2 |  |
| AR | 62.1 | 51.9 | -10.2 | -16.4 | TN | 11.3 | 3.4 | -7.9 | -69.9 | MP | | 30.4 | 27.3 | -3.1 | -10.2 |  |
| PB | 43.3 | 30.7 | -12.6 | -29.1 | JH | 26.5 | 17.8 | -8.7 | -32.8 | WB | | 30.9 | 26.2 | -4.7 | -15.2 |  |
| CG | 49.2 | 36.0 | -13.2 | -26.8 | MN | 17.4 | 8.1 | -9.3 | -53.4 | UK | | 42.5 | 37.0 | -5.5 | -12.9 |  |
| BR | 41.1 | 26.0 | -15.1 | -36.7 | PB | 41.9 | 32.5 | -9.4 | -22.4 | UP | | 40.7 | 33.9 | -6.8 | -16.7 |  |
| RJ | 51.5 | 36.1 | -15.4 | -29.9 | HR | 33.0 | 20.1 | -12.9 | -39.1 | GJ | | 27.0 | 18.7 | -8.3 | -30.7 |  |
| TR | 73.0 | 50.6 | -22.4 | -30.7 |  |  |  |  |  | NL | | 28.8 | 17.8 | -11 | -38.2 |  |
| **India** | **53.4** | **54.8** | **1.4** | **2.6** | **India** | **16.9** | **13.5** | **-3.4** | **-20.1** | **India** | | **25.7** | **26.9** | **1.2** | **4.7** |  |
| ** Proportion of birth outcomes of the last pregnancy in the five years preceding the survey of women age 15-49; AC-absolute changes, RC-Relative changes* | | | | | | | | | | | | | | | | |

| **Additional file 7: Main reasons for abortion and its characteristics among Indian women across the States/UTs during 2019-21 *** | | | | | | | | | | | |  | | |
| --- | --- | --- | --- | --- | --- | --- | --- | --- | --- | --- | --- | --- | --- | --- |
| **States/ UTs** | **Unplanned pregnancies** | **States/ UTs** | **Contraceptive failure** | **States/ UTs** | **Complications in pregnancy** | **States/ UTs** | **Health did not permit** | **States/ UTs** | **Last child too young** | **States/ UTs** | **Foetus had congenital abnormality** | |  |  |
| DL | 73.5 | AR | 12.8 | PB | 26.2 | TN | 30.7 | CG | 24.9 | KL | 16.0 | |  |  |
| TR | 67.9 | MP | 10.7 | JK | 25.9 | TS | 26.9 | OD | 16.1 | AP | 9.7 | |  |  |
| AS | 63.0 | WB | 7.6 | HP | 23.7 | KA | 19.9 | TR | 15.9 | JK | 9.0 | |  |  |
| AR | 62.6 | OD | 6.9 | AP | 22.3 | JH | 19.9 | MN | 14.1 | TS | 8.5 | |  |  |
| UP | 61.3 | DL | 6.3 | KL | 22.1 | JK | 18.7 | WB | 13.2 | GJ | 8.2 | |  |  |
| NL | 56.2 | TR | 5.0 | KA | 18.2 | KL | 17.8 | KA | 12.5 | JH | 7.7 | |  |  |
| WB | 53.8 | HP | 4.8 | TS | 17.7 | AP | 17.6 | NL | 12.4 | PB | 7.6 | |  |  |
| HP | 50.5 | PB | 4.8 | GJ | 17.3 | CG | 15.2 | JH | 11.7 | HP | 7.4 | |  |  |
| OD | 50.5 | KA | 4.8 | MH | 16.9 | UK | 13.5 | KL | 11.5 | KA | 6.6 | |  |  |
| RJ | 49.9 | JK | 4.5 | HR | 14.9 | MH | 13.0 | AS | 10.6 | TN | 6.1 | |  |  |
| BR | 49.8 | UK | 4.3 | RJ | 13.8 | BR | 12.1 | UK | 10.1 | RJ | 6.0 | |  |  |
| UK | 49.3 | RJ | 4.0 | UK | 12.0 | NL | 11.4 | MH | 10.1 | MH | 5.3 | |  |  |
| MN | 48.6 | HR | 3.9 | TN | 8.6 | MN | 11.1 | BR | 9.7 | HR | 4.1 | |  |  |
| GJ | 48.5 | AP | 3.6 | MP | 7.8 | MP | 8.4 | HR | 9.2 | MN | 2.3 | |  |  |
| MP | 48.3 | NL | 3.5 | MN | 6.2 | PB | 8.3 | UP | 8.5 | AR | 2.3 | |  |  |
| JH | 43.0 | MH | 3.4 | UP | 5.5 | WB | 7.9 | AP | 8.3 | MP | 1.7 | |  |  |
| MH | 42.9 | TS | 3.2 | DL | 4.8 | RJ | 7.4 | TS | 8.1 | UP | 1.6 | |  |  |
| HR | 41.4 | JH | 3.2 | JH | 4.7 | HR | 6.9 | TN | 6.9 | DL | 1.2 | |  |  |
| PB | 35.7 | AS | 3.0 | AS | 4.7 | AR | 6.8 | DL | 6.1 | WB | 0.9 | |  |  |
| CG | 32.1 | KL | 2.1 | OD | 4.4 | AS | 6.4 | AR | 5.9 | OD | 0.7 | |  |  |
| TN | 29.8 | GJ | 1.9 | AR | 3.1 | UP | 6.3 | MP | 5.8 | AS | 0.3 | |  |  |
| KL | 17.7 | UP | 1.9 | BR | 2.9 | OD | 6.2 | RJ | 5.5 | BR | 0.3 | |  |  |
| TS | 17.5 | BR | 1.6 | WB | 2.0 | GJ | 6.1 | HP | 4.9 | UK | 0.1 | |  |  |
| JK | 16.9 | CG | 1.0 | TR | 1.9 | HP | 4.2 | JK | 4.6 | CG | 0 | |  |  |
| KA | 16.5 | MN | 0.8 | NL | 1.7 | TR | 2.5 | GJ | 4.2 | TR | 0 | |  |  |
| AP | 14.7 | TN | 0.4 | CG | 1.0 | DL | 2.4 | PB | 1.5 | NL | 0 | |  |  |
| **India** | **47.6** | **India** | **3.6** | **India** | **9.1** | **India** | **11.3** | **India** | **9.7** | **India** | **3.3** | |  |  |
| ** Proportion of birth outcomes of the last pregnancy in the five years preceding the survey of women age 15-49* | | | | | | | | | | | | | |  |
